# Supplementary material for: Modeling post-holiday surge in COVID-19 cases in Pennsylvania counties
Source: PLoS One. 2022 Dec 19;17(12):e0279371. doi: 10.1371/journal.pone.0279371 (PMC9762594; doi:10.1371/journal.pone.0279371)
Supplement: Attachment — Submitted filename: Response to Reviewers.pdf [file pone.0279371.s002.pdf]

Point-by-Point Response to the Reviewer 1

Thank you for carefully reading the paper and for your helpful suggestions. We believe that your feedback regarding additional motivation and context surrounding our methodology, improved the overall quality of our manuscript. We respond below point-by-point to your comments.

Major points:

Comment R1.1

> The first thing to point out is that the problem the authors address is not new. Researchers have been doing this sort of spatial analysis for quite some time. See the books by Lawson (2018), Blangiardo and Cameletti (2015), Moraga (2019), and Banerjee et al. (2015).
>
> Lawson, A. B. (2018). Bayesian disease mapping: hierarchical modeling in spatial epidemiology. Chapman and Hall/CRC.
>
> Blangiardo, M., & Cameletti, M. (2015). Spatial and spatio-temporal Bayesian models with R-INLA. John Wiley & Sons.
>
> Moraga, P. (2019). Geospatial health data: Modeling and visualization with R-INLA and shiny. CRC Press.
>
> Banerjee, S., Carlin, B. P., & Gelfand, A. E. (2015). Hierarchical modeling and analysis for spatial data, Second edition. Chapman and Hall/CRC.

Author Response R1.1

Thank you for bringing additional literature to our attention. We appreciate the suggestion and we have included these references in **Section: Introduction**, third paragraph to provide a more complete background on spatial epidemiology literature (highlighted changes). Additionally, we discussed existing techniques used in epidemiology for spatial temporal data before introducing the motivation for our GEE approach. We also expanded our discussion surrounding the use of GEEs, see **Author Response** R1.4 below.

Comment R1.2

> The solution that most authors apply is the conditional autoregressive (CAR) model, which assumes a covariance structure that depends on the network of neighbors (which can be defined in a number of ways). Data are almost always analyzed in the Bayesian context, using either Markov chain Monte Carlo (MCMC) or the integrated nested Laplace approximation (INLA).

Author Response R1.2

We agree with your comment that CAR models and integrated nested Laplace approximation is widely used in the spatial epidemiology literature. We made the appropriate changes to reflect

these techniques in our background literature. In our revised manuscript we cited Moraga 2019 and Blangiardo & Cameletti 2015 for integrated nested Laplace approximation model for spatial epidemiology. We also cited Zhang et al., 2016 and Blangiardo & Cameletti 2015, as references for the spatial Bayesian CAR model. During our initial internal discussion of the modeling approach, we came across CAR and integrated nested Laplace approximation models but we decided to proceed with a GEE approach due to computation complexity in incorporating spatial correlation and zero-inflation. We outlined the reasoning for our generalized estimating equation (GEE) based method in the manuscript; we added clarifying remarks to highlight these common solutions in spatial epidemiology in the newly added **Section: Introduction**, third paragraph, and introduced the motivation for our GEE approach. See additional discussion of these points in **Author Response** R1.4 below.

Comment R1.3

> The authors propose a model for the covariance matrix that depends on the distances (through the semivariogram). This approach is very close to the concept of kriging, a technique mostly used to interpolate data (such as air pollution measurements) taken at only a few locations across a grid. Predictor variables can be used with kriging, similar to how the authors have used them in the paper under review. The only mention of "kriging" in the paper comes from the article title in reference [36] by Dowd. Lawson (2018), in Section 5.4.2, mentions how a fully specified covariance matrix, like the authors, can be used for areal data, where the measurements are the county-level outcomes at the centroid of the region. Lawson says this approach is "akin to universal kriging."

Author Response R1.3

Your comment regarding the relationship between the semivariogram and kriging raised an interesting point. Our use of a variation of square loss for our semivariogram estimation adds another point of similarity to kriging which defines the square distance through the Gaussian kernel. We included additional comments on kriging in **Section: Introduction**, third paragraph and **Section: Theoretical Semivariogram Model** first paragraph: "…spatial random effects are often modeled using semivariograms which can be viewed as a kriging model in the residual space" to highlight the relationship between semivariograms and kriging. In line with the discussion on robust inference in **Author Response** R1.4, we also selected a robust empirical semivariogram for our model.

Comment R1.4

> The authors' approach uses generalized estimating equations, a classical approach. This is certainly different from the usual approach in the four references described above. Is the authors' approach better than the Bayesian approaches described in the four references above? The answer might be no. But, if the answer is yes, then the paper may be more impactful than the authors realize.

Author Response R1.4

Thank you for bringing this point to our attention. In a related comment, **Author Response** R1.1 & R1.2 described steps taken to contrast our GEE approach with commonly used models in spatial epidemiology. We further expanded on our motivation for GEEs in **Section: Introduction**, third paragraph and **Section: Theoretical Semivariogram Model** first paragraph.

To briefly summarize, we had considered the computational practicality, interpretation and robustness of our proposed GEE when we developed our zero-inflated marginal model. Our GEE follows from a marginal model interpretation where inferences about the population-level effects are the focus while allowing the incorporation of repeated measurements, which are counties in our case. Because in our epidemiolocal setting, our goal is to understand the disease trajectory at a state-level using information from different counties, we proposed GEEs as a flexible alternative that can also incorporate spatial patterns. Zero-inflated GEEs are a special case of mixture of marginal models and have well-established computational strategies for model estimation. These involve implementing an EM-like algorithm and Newton Raphson convex optimization steps. In addition, theoretical motivation of marginal models results in consistent inference even in situations if the covariance structure is misspecified. As a result, even if the spatial correlation is incorrect and we have valid inference through the heteroskedasticity-consistent or robust sandwich estimator. Furthermore, for our GEEs we specify a quasi-likelihood, which then allows us to model over-dispersed Poisson count data which has been shown to be an alternative to negative binomial models in Hall and Zhang 2004. The GEE dispersion parameter accommodates a wide range of situations such as over and under-dispersion. For these reasons, we believe our GEE approach is well justified and included a number of analyses using competing methods to contrast our findings.

Throughout the manuscript, we've added additional clarifying remarks that reflect our previous summary. These highlighted remarks can be found in **Section: GEE for Over-Dispersed Poisson Counts**, second paragraph; **Section: Inference on Coefficients**; and **Section: Discussion.**

Other points:

Comment R1.5

> Be more thorough on lag between contracting COVID and testing positive. There is some science here, especially regarding latency. Local conditions affect time to get tested and reporting lag.

Author Response R1.5
We agree with your assessment and see the value in additional discussion related to latency in case reporting. We designed our analysis to reflect the dynamics of COVID-19 reporting during the beginning of the pandemic. In **Section: Introduction**, first paragraph, we note that during the early pandemic it takes approximately 7-8 days to contract COVID-19 and report a positive case result. We used this information to look at a conservative two-week window from a holiday

to study COVID-19 surge. We discussed additional points regarding local conditions in **Author Response** R1.6.

Due to our analyses regarding the early stages of COVID-19, we look at data from the first year of the pandemic prior to the widespread vaccine distribution, COVID-19 variants and at-home testing. During this time, rigorous disease surveillance and reporting procedures were in place. We include these additional points in **Section: Introduction**, end of the first paragraph.

Comment R1.6

> Be careful with structural zeros. The probabilistic model you suggest may not be reasonable. In my neck of the woods, some health departments never on Saturdays, Sundays, and holidays. Some rarely report on Mondays, presumably because the large backlog takes more than one day. If your model proposes a probability of reporting a zero on a given day, then you may be predicting low probabilities for some counties when the probability should be zero.

Author Response R1.6

You brought up an interesting point for modeling structural zeros. Our EM approach is a classical missing data approach that imputes structural zero labels as the probability of being a structural zero versus a Poisson zero iteratively. The probabilities of structural zeros are used to weigh the quasi-loglikelihood of the GEEs; this becomes the effective sample size of each data point. Counts which are not zero are treated as part of the Poisson model by default. While counts that are zero have mixed membership in either the Poisson or Bernoulli model. When the probability of Poisson model membership goes to zero, the respective log-likelihood weight goes to zero and that data point is removed from the parameter estimation of the count model. You are correct in stating "you may be predicting low probabilities for some counties when the probability should be zero," but these two cases result in very similar parameter estimates and are numerically equivalent. We added this clarifying remark in **Section: GEE for Over-Dispersed Poisson Counts**, first paragraph: "When $w_{i,t} \approx 0$, then the data point is effectively removed from the estimation of Poisson regression parameter." In addition, this reasoning is one of the motivating reasons for including a hurdle model as a competing analysis, which models all zeros separately.

Furthermore, our goal is to model a complex system with heterogeneous data. Unfortunately, we may not be able to capture every facet of heterogeneity of data, but we believe that our GEE strategy is well suited to many situations and zero-inflation. We expand on the previously discussed point in **Section: Introduction**, third paragraph as part of an overall effort to better communicate our GEE mixture model approach (see **Author Response** R1.4).

Comment R1.7

> In the equation at the bottom of p. 3, I expect to see $Y_{i,t-1}$ on the right side. This is, after all, the conditional distribution of $Y_{i,t}$ given $Y_{i,t-1}$. It's not until the next paragraph (on the next page) that we see that mu_i depends on $Y_{i,t-1}$.

Author Response R1.7
Thank you for carefully reading our equations and derivations. We added the conditional form of the $\mu_{i, t} | Y_{i,t-1}$, when we introduce the model in **Section: Zero Inflated Poisson Model** (see the highlighted changes). Later on in the section, we state that we drop the conditional notation in the writing for brevity.

Comment R1.8

> What happens to equation (1) when $y_{i,t-1} = 0$. The log would be undefined. The next equation suggests that the whole expression should be 0.

Author Response R1.8
We've added a clarifying remark in **Section: Zero Inflated Poisson Model,** second paragraph that values must be corrected when y=0. We go on to outline this correction through imputation in **GEE for Over-Dispersed Poisson Counts**.

Comment R1.9

> In Equation (2), the probability on the left conditions on $Z_{i,t-1}$ (cap Z) while the right side involves $z_{i,t-1}$ (lower case z).

Author Response R1.9
In general, we use the uppercase to denote the random variable. We see that this may be confusing when conditioning on past observed values. In our revision, we set the uppercase random variable to the lower case observed value when writing our conditional expectations in equations (1) and (2).

Comment R1.10

> The section on the "Theoretical Semivariogram Model" begins by talking about the residuals. But residuals to what model? There seems to be a Catch-22 here. Residuals from the model are needed to define the model. Is there a way out of this circle?

Author Response R1.10
Thank you for bringing this to our attention; we believe that this also is related to **Comment** R1.3. In our revised manuscript, we use Theoretical Semivariogram Model to introduce our GEE modeling and explain how the residuals may be spatially correlated. In our new **Section: Theoretical Semivariogram Model** first paragraph, we introduce residuals and relate them to semivariograms through mixed models and GEEs. We also motivate GEE estimation as a flexible alternative to mixed models in this section.

Comment R1.11

> p. 3, second last line. This should be "While standard GEEs treat longitudinal data ..."

Author Response R1.11

Thank you for your careful reading of our manuscript. We put forth extra effort into correcting any grammatical errors in our revision.

Comment R1.12

> Figure 1. I don't understand what this plot is showing. The caption describes what the dashed line means, but what are the boxplots on? And shouldn't the dashed lines go through the centers of the boxes? I'm probably missing an important idea, but the caption doesn't help.

Author Response R1.12

Thank you for bringing this point to our attention. We made changes to the manuscript to provide a clear interpretation of the plots. We refer to **Author Response** R2.3 & 2.4 where we address overlapping and similar comments.

We revised the caption of Figure 1: "Box plot of the residual autocorrelations, $Corr(r_{i,t}, r_{i,t-k})$, for lag k (k=1, … 7) from 67 Pennsylvania counties. The residuals $r_{i,t}$ were obtained from a Poisson generalized linear model with study covariates and a lagged autoregressive predictor ($y_{i,t-1}$) as outlined in the COVID-19 data analysis section. Light blue lines connect the autocorrelation values of each county. Solid black line connects the autocorrelation values $Corr(r_t, r_{t-k})$ calculated with residuals from all counties combined"

Comment R1.13

> p. 5, just above the equation in the middle of the page. The colons (:) on the second and first lines above the equation for phi are not needed. Also, in the equation for phi, go ahead and write the indices for i: $\sum_{i=1}^{67}$, or if you want to keep it more general, introduce a variable name for the number of regions and use that.

Author Response R1.13

Thank you for your suggestions; we've made appropriate changes to the grammar and notation. We introduce a set S(t) that i belongs to, S(t) is the set of observed counties at time t (see highlighted changes). Counties report their first case at different dates, resulting in staggered entry into the study; this is easily accommodated by our GEE formulation.

Comment R1.14

> p. 6, line 110. I believe Z_{t-1} should be $Z_{i,t-1}$.

Author Response R1.14

Thank you for your careful reading of our manuscript. We corrected this error in the revised manuscript.

Comment R1.15

> There are exactly S choose 2 unique distances and exactly S choose 2 times T computations of the differences of residuals. Don't interject big O notation if it isn't necessary.

Author Response R1.15
Based on your feedback, we decided to omit the last paragraph in **Section: Empirical Semivariograms** and concisely incorporate the optimization details into the first paragraph of that section.

Comment R1.16
> Be consistent on the citations. Mostly, the authors use the reference number in brackets, e.g. [25], but sometimes they use the name year. At the top of the page, they refer to "Cressie and Hawkins, 1980" which I think should be reference [34]. Later we see Hall and Zhang, 2004.

Author Response R1.16
Thank you for bringing this to our attention. We corrected the reference style and checked that our manuscript follows journal guidelines.

Comment R1.17
> If I'm interpreting Figure 2 right, there are some negative correlations when days since a holiday is small and positive correlations about a week after the holiday. I suspect the former is due to a reporting lag, and the latter is due to the holiday effect. Is this correct? A little more explanation would help here.

Author Response R1.17
We believe this to be the correct interpretation of the results. We did not highlight the negative effects in our initial submission but have added this discussion point to the revision. Please see **Section: Discussion**, second paragraph.

Comment R1.18
> In the last paragraph on p. 13, the authors say there is an IRR of 1.3 to 1.4. It seems to me that this is the number for the state as a whole. What can you say about individual counties? Is this surge higher in some counties than others? Showing maps would be helpful here.

Author Response R1.18
You brought up an interesting point. We address this comment in **Author Response** R1.4, where we explain that GEEs for marginal models result in inference on population-level effects. In this case, these inferences are at the state level. Because we do not model county-level random effects as we would in mixed models, we no longer have county-specific inference. This is one of the tradeoffs of marginal models versus mixed models: we gain flexibility in parameterization and reduce the complexity of computation at the expense of county-specific inference.

Comment R1.19

> In line 361, the authors say "All but the hurdle model, estimates in Figure 2 confirm a drop in the case counts on a holiday, with all the backlogged cases being reported the next day." I think it is optimistic to say that ALL backlogged cases are reported the next day. Around here, this is certainly not the case.

Author Response R1.19

You raised a valid concern and we agree that the "all the backlogged cases being" is strongly worded. We made the appropriate changes to **Section: Discussion**, third paragraph: "… with many of the backlogged cases being reported the next day, evident by a large positive effect a day after a holiday."

Comment R1.20

> The section on Supporting Information seems to be blank.

Author Response R1.20

We've updated the supporting information section with the appropriate funding information.

Comment R1.21

> Be consistent in capitalizing journal names. Either capitalize the first letters of important words always or never. For example, we have "New England journal of medicine" but "International Journal of Infectious Diseases." On p. 16, we see "American journal of public health" and "Journal of the American Statistical Association." Also, "Jama" looks odd.

Author Response R1.21

We corrected the reference style and checked that our manuscript follows journal guidelines.

Point-by-Point Response to the Reviewer 2

Thank you for many useful suggestions regarding study motivation and methodology. These points helped improve the readability and quality of our manuscript. We respond below point-by-point to your comments.

Comment R2.1

> General comment: In this paper, the authors propose to use the zero-inflated Poisson model with spatial correlation to model the COVID-19 case count. Through including time-varying holiday covariates, the model is used to estimate the effect of holiday timing on case count. After applying the proposed method to the COVID-19 case counts in Pennsylvania counties, the authors find that there is a surge in COVID-19 cases approximately one week after a holiday. The introduction of the proposed method is clear. However, there are some points that are not reasonable or need to be further explained. Since these data were obtained before widespread vaccine distribution, now the vaccine is widely distributed in many countries, the conclusions of this research problem do not have high guiding value for the actual situation.

Author Response R2.1

Thank you for bringing these points to our attention. We believe that there is merit to understanding early pandemic dynamics. We began planning our analysis in 2020; we've realized that our writing may reach an audience far removed from the beginning of the pandemic possibly with vaccines and other treatment options available. Infection data from the early pandemic, collected according to rigorous reporting procedures is a rare and invaluable resource for epidemiological studies. In addition, our modeling approach is applicable to future pandemics and many different epidemiological settings. We revised our manuscript to contextualize the early pandemic with respect to our analysis, see **Section: Introduction**, end of the first paragraph, and **Section: Discussion**, last paragraph (highlighted changes). We believe that our findings and modeling approach are applicable to a wide range of epidemiological settings.

In addition, based on feedback from Reviewer 1, we included additional motivation for the marginal model and generalized estimating equations (GEE) approach. In **Section: Introduction**, third paragraph, we added a thorough review of existing methods and explain the utility of GEEs. Among these, we motivate our GEE approach by the ability to handle over-dispersion, zero-inflation, spatial correlation, and robust inference under misspecified spatial correlation.

Comment R2.2

> Can you explain why do you choose to use a Poisson model to model the case count, not other models which also fit for discrete variables?

Author Response R2.2

We proposed a generalized estimating equation (GEE) based method with a dispersion parameter to handle situations with over-dispersion. Our GEE approach accommodates over-dispersion much like a negative binomial regression if over-dispersion is present (Hall and Zhang 2004). We outline our rationale for the mixture of marginal models approach in **Author Response** R1.4. In addition to accommodating over-dispersion, the Poisson GEEs are a starting point for us to incorporate spatial modeling in the correlation structure and zero-inflation as well as providing flexible procedures for the optimization and inference of model parameters.

Comment R2.3

> In the Poisson regression model (1), only the case count from the previous day is used to account for the temporal correlation of counts in the same county, is it enough? Since the virus has an incubation period, the case count in one day may depend on the case counts several days ago.

Author Response R2.3

You brought up an important point. During the planning of the analysis, we discussed internally how to proceed with the autoregression component of our model. This problem was further compounded by the presence of structural zeros or zero-inflation in the count data. We performed the model diagnostic on a simple GLM (Figure 1), a Poisson regression with the same study covariates. We found little autocorrelation in the residuals (Figure 1) from within the same county, which suggests that a 1 lag difference autoregression is adequate for analysis.

We want to emphasize that under the simple GLM setting, which does not account for zero-inflation, we've already observed low autocorrelation and the inclusion of a zero-inflation mixture model was a crucial component of our approach. The zero-inflation, structure zero label imputation (outlined in **Author Response** R1.6), would further decrease the already low autocorrelation of the residuals. When the model estimates that a zero count is a structural zero with high probability, it removes that observation from the Poisson GEE model. We believe that compounding the weak autocorrelation observed from a naïve model and in combination with mixture modeling effectively addresses autocorrelation. In addition, we believe that holiday and weekend timing to be the major driver of temporal patterns in our analysis, with case reporting being impacted by holidays and weekends based on dataset guidelines. Therefore, we believe that 1 lag count predictors are sufficient for our analysis.

We expanded the explanation of our reasoning with clarifying remarks in the manuscript **Section: Theoretical Semivariogram Model**, third paragraph: "Fig 1 shows low autocorrelation of residuals, $Corr(r_{i,t}, r_{i,t-k})$, within each county from a naive standard Poisson generalized linear model described by equation (1) using a lagged autoregressive predictor ($y_{i,t-1}$) and study covariates while assuming no zero-inflation. Our implementation of zero-inflated modeling assumes some observations belong to a structural zero model which further decreases autocorrelation in residuals, suggesting a single lag autoregressive predictor adequately addresses autocorrelation in the residuals. Furthermore, we expect the structural zeros to be related to holiday and weekend timings based on dataset guidelines and case

counts to be cumulatively reported after a holiday. As a result, we anticipate holiday and weekend timings to be the major driver of temporal case trajectories in our study.”

Comment R2.4

> For the proposed approach, it is said that dates are treated as independent clusters, and counties are treated as repeated measures in the GEE formula. Related with the previous question, if only the case count from the previous day is used to account for the temporal correlation of counts in the same county, it may not enough to eliminate all correlations among residuals from different days. From Figure 1, it can be found that for some lags, such as lag 1 and lag 7, the autocorrelation of residuals is not low.

Author Response R2.4

Thank you for your comments regarding Figure 1. We acknowledge additional clarification surrounding the residuals is needed and we appropriately revised **Section: Theoretical Semivariogram Model**, third paragraph and Figure 1 caption accordingly.

We detailed our rationale for the 1 lag autoregression in **Author Response** R2.3. We do observe a slight increase in lag 7 autocorrelation. This was likely due to the effects of the remaining day of the week even after accounting for the day of the week as a covariate. However, the 7 lag autocorrelation remains low and for reasons outlined in **Author Response** R2.3 we opt to use a 1 lag autoregression. A 7 lag autoregression would over parameterize the model without much gain in reducing autocorrelation.

Autocorrelation diagnostics for our time-series mixture models are less common and difficult to interpret. In essence, each residual now has an imputation weight associated with it. In cases where weights are near 0 or 1 equates to a high likelihood of belonging to the structural zero or Poisson count group. However, when weights are mixed, e.g., near 0.5, then they can be interpreted as belonging to both groups. This is advantageous for parameter estimation as it allows the uncertainty to be propagated in both the Poisson and logistic regression. The interpretation of residuals associated with 0 weights i.e., missing data (**Author Response** R1.6) are less straightforward when calculating autocorrelations.

Comment R2.5

> For the real data, the data are obtained before the widespread vaccine distribution. But now the vaccine is widely distributed in many countries and the incubation period for COVID-19 has become much shorter, the findings cannot directly guide practice, so the application value of this research problem is not very high.

Author Response R2.5

Please refer to **Author Response** R2.1 where we address overlapping and similar comments.

Comment R2.6

> Regarding the holiday covariate, only one day off is considered for a holiday. However, for some holidays, the number of days off is more than one day, such as New Year and Christmas. So the holiday covariate cannot reflect the actual situation. Furthermore, the results can be biased.

Author Response R2.6

To clarify, we take the holiday timing and transform them into a vector of length 15 to account for holiday timing effects regardless of the number of days off for a given holiday. Elements of the vector are ordered to reflect days since a calendar holiday date. From the second paragraph of the **Poisson model covariates** section: “For example, the count recorded on January 1st, the associated 15 holiday lag indicators are (1, 0, 0, 0, 0, 0, 0, 1, 0, 0, 0, 0, 0, 0, 0) for dates: New Years, Dec 31, Dec 30, Dec 29, Dec 28, Dec 27, Dec 26, Christmas, . . . , Dec 18. We abbreviate the indicator for a holiday being k days in the past as, Holiday Lag k: HLk. Another example, for the count data on January 2nd, the indicators are (0, 1, 0, 0, 0, 0, 0, 0, 1, 0, 0, 0, 0, 0, 0).”

This vector allows us to study a conservative 2-week timing after a holiday date. If one contracts COVID-19 during a one-day off holiday, then we expect a case report 7-8 after the calendar date. If it’s a Monday holiday such as, Memorial day, one travels contracts COVID-19 on Friday or Saturday, we expect the spike in case reports to be 5-6 after the holiday calendar date. Our 15 dimension vector can also handle the case when the holidays overlap within a 2-week period, such as Christmas and New Year (see **Section: Poisson Model Covariates**, second paragraph) to consider the timings of these two holidays are considered simultaneously, using multiple indicators. As noted in **Section: Poisson Model Covariates**, second paragraph, the indicators shift to the right in the array when the date associated with the data moves past a holiday. A surge occurs 6-8 days after a holiday; we observed a positive effect for the 6-8 day lag predictors, a 3-day window where we observe elevated cases. This 3-day window reflects the uncertainty surrounding the exact holiday date timings (see **Section: Discussion**, second paragraph).

One of the motivating factors for using GEEs with robust sandwich estimators and robust semivariograms (**Author Response** R1.3 & R1.4) is to address the issue of bias. Confidence intervals produced with sandwich estimators are considerably wider i.e., more conservative, than treating the data as iid. This is also why we applied the robust estimator to GLM estimates and other competing methods.

Comment R2.7

> It is said that the precipitation and temperature are further transferred into binary variables. How are these binary variables defined?

Author Response R2.7

For the temperature and precipitation lag effects, only precipitation was binarized based on whether it rained or not on that day. The variable for temperature was left as a continuous

predictor. We revised **Section: Poisson Model Covariates**, third paragraph to make this distinction clear.

Comment R2.8

> It is found that a surge in case counts occurs about 6-8 days after a holiday. Can you explain the reason for this?

Author Response R2.8

Our findings are consistent with the other scientific evidence and we amended **Section: Discussion**, second paragraph with: “This is in line with the anticipated timing of 4–5 days to symptom onset and up to 3 days for PCR testing results.”

Comment R2.9

> In the Poisson model, many covariates are considered. But some of the covariates are highly correlated, such as poverty rate, log median house value, and log median household income. It may cause the multicollinearity problems.

Author Response R2.9

You brought up an interesting point. When we planned the analysis, we look at well-known but similar analyses for guidance such as Wu et al. 2020 and Kontis et al. 2020. We cite Wu et al. 2020 which used the same demographic covariates as our study, in addition to other covariates. Furthermore, we did not encounter signs of multicollinearity in our model diagnostics. Among these signs, include inflated standard errors and issues with the convergence of the optimization algorithm.

Comment R2.10

> It is found that counties with a high rate of owner occupied housing were significantly associated with an increased probability of reporting zero cases in both models. Then the authors explain that it may because rural counties may have more logistical challenges in reporting cases at a daily frequency. Is it possible that because rural counties have less population densities than urban counties, and the COVID-19 virus is less likely to spread in rural counties?

Author Response R2.10

Thank you for bringing this to our attention. We believe this to be a plausible explanation and amended **Section: COVID-19 Data Analysis**, with this interpretation.

Comment R2.11

> It says “After the estimations have converged, we used the robust covariance estimator, which is consistent under misspecification, to compute standard errors.” Here, it needs to be explained which kind of misspecification it is.

Author Response R2.11

Thank you for raising this point. In our revision, we added clarifying comments on the inferential procedure that takes place with GEEs and sandwich estimators in **Section: Inference on Coefficients**: "…we have consistent estimates even under misspecified working correlations which are spatial correlations in our case. We proposed a reasonable spatial correlation model but retain reliable inference even in situations when the correlation model is incorrect."

References

Hall DB, Zhang Z. Marginal models for zero-inflated clustered data. Statistical Modelling. 2004;4(3):161–180.

Zhang, L., Baladandayuthapani, V., Zhu, H., Baggerly, K., Majewski, T., Czerniak, B. & Morris, J. Functional CAR models for large spatially correlated functional datasets. Journal of the American Statistical Association. 2016;111(514), 772-786

Wu X, Nethery R, Sabath M, Braun D, Dominici F. Air pollution and COVID-19 mortality in the United States: Strengths and limitations of an ecological regression analysis. Science advances. 2020;6(45):eabd4049.

Kontis V, Bennett JE, Rashid T, Parks RM, Pearson-Stuttard J, Guillot M, et al. Magnitude, demographics and dynamics of the effect of the first wave of the COVID-19 pandemic on all-cause mortality in 21 industrialized countries. Nature medicine. 2020; p. 1–10.
