# Supplementary material for: Modeling post-holiday surge in COVID-19 cases in Pennsylvania counties
Source: PLoS One. 2022 Dec 19;17(12):e0279371. doi: 10.1371/journal.pone.0279371 (PMC9762594; doi:10.1371/journal.pone.0279371)
Supplement: Attachment — Submitted filename: Referee report.pdf [file pone.0279371.s001.pdf]

Referee report on “Modeling Post-Holiday Surge in COVID-19 Cases in Pennsylvania Counties”

General comment: In this paper, the authors propose to use the zero-inflated Poisson model with spatial correlation to model the COVID-19 case count. Through including time-varying holiday covariates, the model is used to estimate the effect of holiday timing on case count. After applying the proposed method to the COVID-19 case counts in Pennsylvania counties, the authors find that there is a surge in COVID-19 cases approximately one week after a holiday. The introduction of the proposed method is clear. However, there are some points that are not reasonable or need to be further explained. Since these data were obtained before widespread vaccine distribution, now the vaccine is widely distributed in many countries, the conclusions of this research problem do not have high guiding value for the actual situation.

Comments for the authors

1. Can you explain why do you choose to use a Poisson model to model the case count, not other models which also fit for discrete variables?

2. In the Poisson regression model (1), only the case count from the previous day is used to account for the temporal correlation of counts in the same county, is it enough? Since the virus has an incubation period, the case count in one day may depend on the case counts several days ago.

3. For the proposed approach, it is said that dates are treated as independent clusters, and counties are treated as repeated measures in the GEE formula. Related with the previous question, if only the case count from the previous day is used to account for the temporal correlation of counts in the same county, it may not enough to eliminate all correlations among residuals from different days. From Figure 1, it can be found that for some lags, such as lag 1 and lag 7, the autocorrelation of residuals is not low.

4. For the real data, the data are obtained before the widespread vaccine distribution. But now the vaccine is widely distributed in many

countries and the incubation period for COVID-19 has become much shorter, the findings cannot directly guide practice, so the application value of this research problem is not very high.

5. Regarding the holiday covariate, only one day off is considered for a holiday. However, for some holidays, the number of days off is more than one day, such as New Year and Christmas. So the holiday covariate cannot reflect the actual situation. Furthermore, the results can be biased.

6. It is said that the precipitation and temperature are further transfered into binary variables. How are these binary variables defined?

7. It is found that a surge in case counts occurs about 6-8 days after a holiday. Can you explain the reason for this?

8. In the Poisson model, many covariates are considered. But some of the covariates are highly correlated, such as poverty rate, log median house value, and log median household income. It may cause the multicollinearity problems.

9. It is found that counties with a high rate of owner occupied housing were significantly associated with an increased probability of reporting zero cases in both models. Then the authors explain that it may because rural counties may have more logistical challenges in reporting cases at a daily frequency. Is it possible that because rural counties have less population densities than urban counties, and the COVID-19 virus is less likely to spread in rural counties?

10. It says "After the estimations have converged, we used the robust covariance estimator, which is consistent under misspecification, to compute standard errors." Here, it needs to be explained which kind of misspecification it is.
